# Rhizospheric Precipitation of Manganese by Phosphate: A Novel Strategy to Enhance Mn Tolerance in the Hyperaccumulator *Phytolacca americana*

**DOI:** 10.3390/toxics11120977

**Published:** 2023-12-01

**Authors:** Changming Dou, Cuicui Qi

**Affiliations:** Anhui Provincial Academy of Eco-Environmental Science Research, Hefei 230061, China; doucm@sina.com

**Keywords:** exclusion, manganese, phosphate, pokeweed, resistance, rhizosphere precipitation

## Abstract

Manganese (Mn) exclusion in the Mn hyperaccumulator pokeweed (*Phytolacca americana* L.) was investigated. Hydroponic experiments were carried out to observe the responses of pokeweeds continually exposed to high levels of Mn. In this study, crystals were observed to appear firstly on the root hair, and soon after, more crystals appeared on the root surface, and crystals of Mn phosphate were observed to appear on the root surface in a time sequence negatively correlated with the number of leaves treated with 5 mM Mn. Crystals were identified via phase analysis of X-ray diffraction and element analysis, and these white insoluble crystals were identified using XRD to be Mn phosphate, with the molecular formula (Mn,Fe)_3_(PO_4_)_2_·4H_2_O. The nutrient solution pH increased from 4.5 to about 5.6 before the crystals appeared. Mn phosphate crystals appeared in all solutions except those without phosphate and emerged earlier in the solutions containing no Fe. Compared with control group, pokeweed accumulated much more Mn in the leaves when treated without phosphate or Fe. The present study suggests that pokeweed can exclude Mn by means of rhizosphere precipitation by phosphate to form Mn phosphate crystals that accumulate on the root surface. Although the detailed mechanism requires further investigation, this study provides the first direct evidence of a novel strategy to inhibit Mn uptake in the roots of a hyperaccumulator in a P-enriched environment.

## 1. Introduction

Manganese (Mn) and aluminum (Al) toxicity and phosphorus deficiency in acid soils are three of the most important factors that significantly limit crop production worldwide [1]. A broad spectrum of research at different levels has been carried out, and great progress has been made in the understanding of the potential mechanisms underlying plant resistance to Al phytotoxicity [2,3]. However, the knowledge of Mn resistance in plants remains lacking. Additionally, phytoremediation, which is green and has a low carbon output compared with other methods, is an effective treatment for heavy-metal-contaminated soil. For a long time, researchers have screened plant species that can be used for phytoremediation and achieved certain remediation effects [4,5,6]. However, the mechanisms underlying the tolerance to and accumulation of heavy metals in these phytoremediation plants are still unclear.

Only in the last decade have Mn toxicity and detoxification in plants received more attention, with some Mn hyperaccumulators being discovered [7,8,9,10,11]. Studies have been concentrated on investigating Mn accumulation, distribution, translocation and speciation in some hyperaccumulating plants to gain insight into the mechanisms of Mn tolerance or internal detoxification. In addition, some genes involved in Mn transport and tolerance in plants were identified and further confirmed at the molecular level [11,12,13,14,15,16]. To date, Mn tolerance in plants is largely assumed to be similar to Al tolerance mechanisms, which mainly include chelation by organic acids and compartmentation in the vacuole or cell wall.

Heavy metal resistance can usually be divided into mechanisms conferring on plants the ability to tolerate metal in the plant symplast (metal tolerance) and mechanisms preventing metal uptake or facilitating metal exclusion from the root (metal exclusion) [17]. Up to now, there has been little research on the mechanisms of Mn exclusion in plants. Taking the chemical and physiological properties of Mn into consideration, possible measures which could be taken by plant roots to exclude Mn are hypothesized as follows: (1) release oxygen and oxidate the freely reactive form of Mn (Mn^2+^) into the unavailable forms (Mn oxides, usually MnO_2_); (2) secrete some organics that contain effective ligands to chelate Mn, such as organic acids and phenolic compounds; (3) immobilize Mn in the rhizosphere by means of inorganics, for example, phosphate; and (4) elevate rhizosphere pH and thus reduce Mn activity.

Rhizodeposition of heavy metals by phosphate could be an effective mechanism by which plants to exclude heavy metals. However, convincing evidence in support of this mechanism has not been presented. Phosphate has been reported to reduce the toxicity of excess heavy metals, such as Al, Pb, Zn, Mn and Cd, by rendering them inactive within plants or via precipitation within plant roots [18,19,20,21,22,23,24]. It has been previously reported that phosphorus was detected in the root apical exudates of an Al-tolerant maize cultivar exposed to Al, which indicates that Al-triggered phosphorus exudation from the root apex might be an important component of Al tolerance in maize, because the released phosphorus could precipitate Al as Al-phosphate in the root cell wall [25]. Later, Zheng et al. (2005) suggested that the greater Al resistance in buckwheat was related to the immobilization and detoxification of Al by phosphorus in the root tissues [20]. These studies and relative research indicated the possible precipitation effect of phosphorus in roots, but no direct evidence of the precipitation of Al, or other heavy metals, by phosphorus in the form of phosphate was provided.

Mn phytotoxicity is different from that of Al, and it is primarily expressed in the shoot, typically being characterized by chlorosis and necrotic lesions in the leaves. Former studies on Mn resistance in plants focused more on the shoot and less on the root. Pokeweed (*Phytolacca americana* L.) is a Mn hyperaccumulator that has received much attention recently [7,9,10,26,27,28]. In a previous study, we reported the potential mechanisms of Mn internal detoxification in leaves [7]. The plant exhibited a great ability to tolerate internal and external Mn and showed no discernable phytotoxic symptoms with extensive Mn accumulation. However, the internal accumulation was limited, and the resistance of pokeweed to Mn cannot be explained only by mechanisms of internal detoxification. So, one interesting question is how the plant responds to continual exposure to high levels of Mn after sufficient accumulation. Pokeweed must possess some mechanisms to exclude Mn by the root, and the root must perform some function to contribute to the resistance to Mn of the plant. During our research on pokeweed, we found that phosphate could be closely related to Mn exclusion. In the present study, we investigated Mn exclusion mechanisms in pokeweed under hydroponic conditions and provided direct evidence for the mechanism of rhizosphere deposition by phosphate in plants.

## 2. Materials and Methods

### 2.1. Plant Materials and Growth Environments

Pokeweed (*Phytolacca americana* L.) was collected from the Mn tailing area of Hunan Province, China. Seeds were soaked in deionized water overnight and then germinated in a plastic tray filled with sand at 25 °C in the dark. The seedlings at the four-leafed stage were transferred and grown hydroponically in a greenhouse equipped with supplementary lighting in a 14 h photoperiod at conditions of 25 °C day/20 °C night and a relative humidity of 70%. Hoagland’s nutrient solution was used, which consisted of Ca(NO_3_)_2_·4H_2_O (5 mM), KNO_3_ (5 mM), MgSO_4_·4H_2_O (2 mM), KH_2_PO_4_ (1 mM), EDTA-Fe (0.1 mM), H_3_BO_3_ (46 μM), MnCl_2_·4H_2_O (5 μM), CuSO_4_·5H_2_O (0.32 μM), ZnSO_4_·7H_2_O (0.76 μM) and Na_2_MoO_4_ (0.5 μM). After a 30-day preculture in 1/2-strength Hoagland’s nutrient solution, uniform seedlings were selected and transplanted into 1.2 L plastic pots (one seedling per pot) for different treatments. Each treatment was replicated three times. The solutions were adjusted to pH 4.5 with 0.1 M HCl or 0.1 M NaOH initially and renewed every two days. In addition, chemical speciation calculations with GEOCHEM-PC indicated no precipitation and little complexation of cations with anions in all the solutions.

### 2.2. Observation of Mn Exclusion in Pokeweed

As the leaf is the most important storage organ for Mn, we wanted to know how the plants would respond to continual exposure to high levels of Mn after sufficient accumulation. Uniform seedlings were randomly divided into three groups treated with 5 mM Mn, including (1) a normal control, (2) a group with about one third of the leaves cut and (3) a group with about one third of the leaves left. The treatment concentration of Mn chosen for this study was based on a previous study [4]. The nutrient solutions with an initial pH of 4.5 were renewed every two days but not aerated throughout the experiment, and the pH was measured every 24 h with a pH meter (Leici PHS-25, Shanghai, China). Special attention was paid to the plant roots and solutions to observe whether any crystals or precipitations appeared on the root surface or in the solutions, and if so, to collect them for further analysis.

### 2.3. Phase Analysis of X-ray Diffraction (XRD)

Crystals on the root surface were collected and washed with deionized water several times and then freeze-dried in a freeze drier (Christ Alpha 1-4, Osterode, SN, German) for X-ray diffraction analysis. The X-ray powder diffraction spectra were collected on a Rigaku D/Max-2500 powder diffractometer (Rigaku, Tokyo, Japan) using Cu Ka (λ for Ka = 1.54059 Å) radiation at 40 kV and 250 mA. The scans were run from 3.0 to 70.0° 2θ, with an increasing step size of 0.02° and a 5°/min of scan rate. Data were processed using the MDI-Jade version 7.0 software (Materials Data Inc., Livermore, CA, USA).

### 2.4. Effect of the Initial Solution pH on the Formation of Mn Crystals

Pokeweed seedlings were also treated with 5 mM Mn, but with an initial pH of 5.6. Other treatments were carried out as described above. We aimed to assess whether and when Mn crystals would appear.

### 2.5. Effect of External Phosphate and Iron (Fe) Deficiency on Mn Exclusion in Pokeweed

To further assess the effect of external phosphate and Fe on Mn exclusion in pokeweed, three groups of pokeweed seedlings were exposed to 5 mM Mn with different treatments, including (1) normal control, (2) –P, with no phosphate in the nutrient solutions and (3) –Fe, with no Fe in the nutrient solutions. Other conditions were consistent with the aforementioned method. The appearance of crystals was attentively watched during the treatment. The accumulation of Mn in mature leaves was investigated on d 10, and pokeweeds were further cultured and observed.

### 2.6. Elemental Analysis

Elements of the above crystals were further determined via element analysis and inductively coupled plasma atomic emission spectrometry (ICP). C, H and N were determined through element analysis (Flash EA 1112, Thermofisher, Monza, Italy). Other mineral elements were detected using ICP (IRIS/AP optical emission spectrometer, Thermo Jarrel Ash, San Jose, CA, USA).

The Mn concentrations of plant samples were determined via flame atomic absorption spectrometry (AAS). All plant samples were wet-digested with a mixture of HNO_3_ and HCl (3:1) and then with HClO_4_. All concentrations of Mn in the digests were determined via AAS (Solaar MK II M6, Thermo Elemental, Boxford, MA, USA). In metal analysis, the analysis method is verified with relevant standard reference materials, and the recovery rate is about 90–104%.

### 2.7. Statistic Analysis

The statistics software package SPSS 13.0 (SPSS Inc., Chicago, IL, USA) was employed to process the data. Analysis of variance (ANOVA) was performed on the data sets. *p* values of less than 0.05 were considered statistically significant.

## 3. Results

### 3.1. Observation of Mn Exclusion in Pokeweed

**Figure 1:**
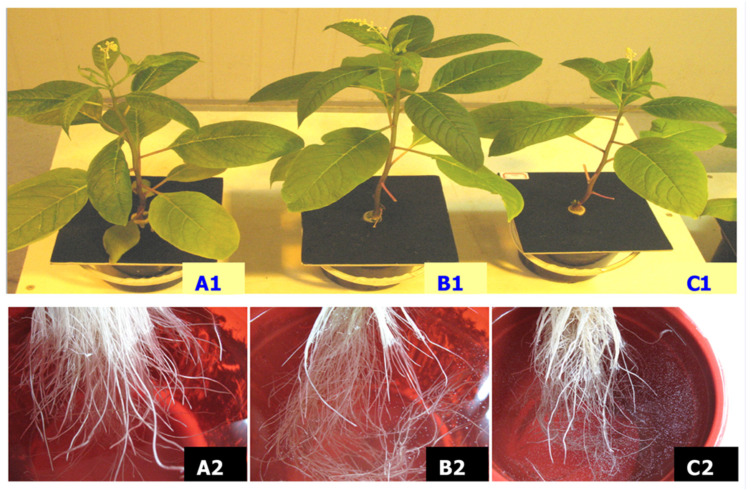
Responses of pokeweeds and roots continually exposed to 5 mM Mn for 6 d. (**A1** and **A2**), the leaves and roots of control, respectively; (**B1** and **B2**), the leave and roots of treatment with about one third of the leave cut, respectively; (**C1** and **C2**), the leave and roots of treatment with about one third of the leaves left, respectively.

**Figure 2:**
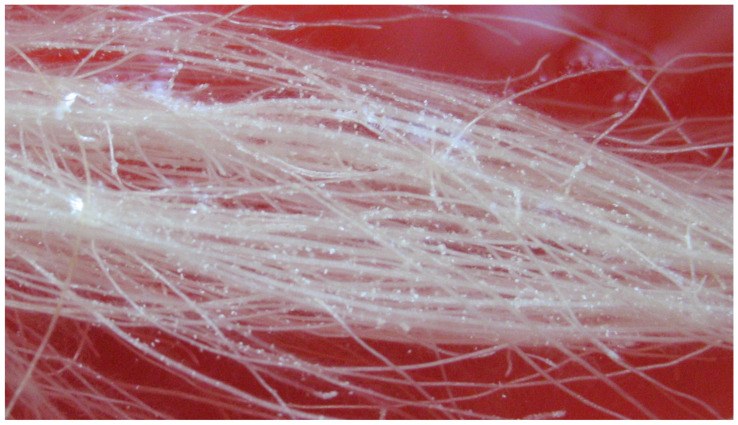
Close-up of the crystals on the pokeweed root surface.

Three groups of pokeweeds with different numbers of leaves were treated with 5 mM Mn (Figure 1). We found that, on d 5, pokeweeds with the fewest leaves were the first to generate white crystals on the root hair and root surface (Figure 1(C1,C2) and Figure 2) and that plants with more leaves had crystals later (Figure 1(A1,A2,B1,B2)). The crystals were observed to appear firstly on the root hair, and soon after, more crystals appeared on the root surface (Figure 2). With the crystals growing and accumulating, they came off the root and floated on the surface of the nutrient solutions or were deposited in the solutions.

### 3.2. Identification of the Crystals

**Figure 3:**
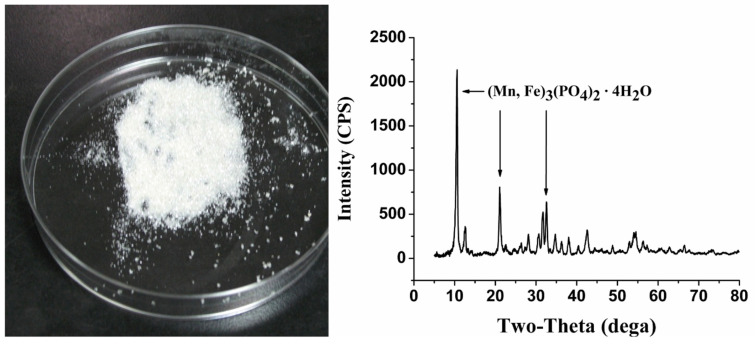
Identification of the crystals via phase analysis X-ray diffraction.

These white insoluble crystals were identified using XRD to be Mn phosphate (Figure 3), also called metaswitzerite, with the molecular formula (Mn,Fe)_3_(PO_4_)_2_·4H_2_O, or usually Mn_3_(PO_4_)_2_·4H_2_O. The results of XRD were further verified via element analysis, wherein it was found that the elements C and N were not present, while concentrations of 1.9% H, 37.6% Mn, 14.5% P and 0.9% Fe were present in the crystals.

### 3.3. Effect of the Nutrient Solution pH on the Formation of the Crystals

The pH of all the above nutrient solutions increased before Mn crystals appeared. It was noted that the pH of all the nutrient solutions increased to nearly the same value, about 5.6, and crystals were found in every pot on d 8. Then, the renewed solution pH increased quickly from 4.5 to about 5.8, and soon after, Mn crystals appeared on the root surface. The findings of the appearance of Mn crystals and the increase in the solution pH were also observed when the plants were treated with different concentrations of Mn^2+^ from 1 mM to 5 mM, and Mn crystals appeared in a time sequence positively correlated with the treatment concentrations of Mn^2+^.

The solution pH seemed to be an important factor for the appearance of Mn crystals. However, Mn crystals were still observed to appear after 6 to 7 d, when pokeweeds were exposed to 5 mM Mn with an initial pH of 5.6.

### 3.4. Effect of Phosphate and Fe Deficiency on the Formation of Mn Crystals and Mn Toxicity in Pokeweed

Mn phosphate crystals were found on the root surface in all solutions except those without phosphate, and the crystals appeared earlier in the solutions containing no Fe. Compared with the control group, pokeweed accumulated much more Mn in its leaves when treated without phosphate or Fe (Table 1). After further culturing, typical symptoms of Mn phytotoxicity, such as the necrosis of young leaves and chlorosis in leaves, were soon after (about on d 15) found in pokeweeds treated with no phosphate, while no toxic symptoms were observed in the plants of control group.

## 4. Discussion

Much attention has been drawn to phosphate’s rhizosphere precipitation of heavy metals recently. Several plants have been reported to secret phosphate when exposed to heavy metals, and a significant increase in phosphate and heavy metals was found within plant roots [20,21,25]. Mn toxicity was also reported to be reduced through rhizodeposition and by rendering it inactive in plants [29]. Generally, insoluble heavy metal phosphate precipitates are considered nontoxic to plants; however, they can accumulate on the root surface, in the cell wall, or in root cells and clog the root pores to prevent the uptake of phosphate and other essentials [30,31,32]. The present study also found that external phosphate could retard the uptake of Mn into pokeweed (Table 1) and reduce or eliminate Mn phytotoxicity. The leaf is the main storage organ of Mn accumulation in a plant. Pokeweeds with fewer leaves were observed to generate Mn crystals earlier (Figure 1), which suggests that the plant could renew its efforts to resist Mn after taking up Mn to a certain degree. Moreover, Mn phosphate crystals accumulating on the root surface are direct evidence of the exclusion mechanism of Mn rhizodeposition by phosphate (Figure 1, Figure 2 and Figure 3).

Mn toxicity is one of the most important limiting factors of acid soils [33]. Therefore, it is of special importance for plants in these areas to elevate their rhizosphere pH. The pH of all the studied treatment solutions containing phosphate was observed to increase significantly before Mn phosphate crystals appeared when pokeweeds were exposed to Mn. Although the solution pH is different from the rhizosphere pH, the changes in the solution pH can still reflect the rhizosphere pH to a great extent under a hydroponic system. The present results indicate that the solution pH, or the rhizosphere pH, could be a prerequisite to the accumulation of Mn phosphate crystals on the root surface.

Crystals accumulated on the root surface were identified to be metaswitzerite, (Mn,Fe)_3_(PO_4_)_2_·4H_2_O. Fe seems to play an important role in crystal formation. However, that is not the case. Further experiments showed that pokeweed did accumulate more Mn in the leaves when treated without Fe (Table 1), but the crystals appeared regardless of the nutrient solutions used or whether there was any Fe. In addition, the empirical formula of switzerite is (Mn_0.97_Fe_0.03_)_3_(PO_4_)_2_·7H_2_O, and Fe is usually present at negligible levels, which was also confirmed through elemental analysis.

Considering all the present results, Mn resistance in pokeweed through rhizodeposition could result rationally from the interaction of Pi and rhizosphere pH. The formation of Mn phosphate crystals relies on Mn^2+^, Pi and a certain pH in the rhizosphere. It has been found that root Pi uptake is accompanied by H^+^ influx, and hence leads to alkalization of the media [34]. The present findings agree well with this conclusion. It is very likely that for pokeweed continuously exposed to high levels of external Mn, with the uptake of Pi, Mn and other nutrients, the plant firstly accumulates sufficient Mn within its tolerance capacity, the rhizosphere pH increases simultaneously, and then pokeweed triggers new resistance mechanisms to exclude Mn, in which Mn can be precipitated by phosphate to form Mn phosphate crystals that accumulate on the root surface. However, one question necessitating further research is where the element Mn in the crystals comes from: the nutrient solution or the plant.

Numerous studies have suggested that hyperaccumulators can enhance the bioavailability of target metals through various rhizosphere behaviors. Previous research has clearly indicated that organic acids secreted by pokeweed roots activate Mn in the soil, promoting its uptake and accumulation. However, this phenomenon predominantly occurs in phosphorus-deficient soil [28]. In contrast, our results suggest that pokeweed employs a distinct adaptation strategy, resulting in the formation of Mn–phosphate crystals in the rhizosphere, even in environments with a comparable concentration of phosphorus. This observation implies that the accumulation of Mn in pokeweed shoots serves as a detoxification mechanism, involving the translocation of Mn from the roots to the shoots. If the activity of Mn in the root system can be effectively restricted, there would be no need for its transfer to the shoots.

Given the generally low availability of phosphorus in soil, detoxifying manganese in the rhizosphere by forming Mn-phosphate crystals is not feasible. Therefore, it is possible that pokeweed has evolved to have an enhanced capacity for manganese transportation to the ground, contributing to its status as a manganese hyperaccumulator. Our results uncover a novel mechanism of plant manganese detoxification in P-abundant environments, providing an in-depth understanding of the metallicity tolerance mechanisms in hyperaccumulators. This further substantiates the relationship between phosphorus, a crucial mineral element, and heavy metal accumulation, providing valuable insights for soil remediation practices using hyperaccumulators.

## 5. Conclusions

In this study, three groups of pokeweeds with different numbers of leaves were treated with 5 mM Mn, finding that on d 5, pokeweeds with the fewest leaves were the first to generate white crystals on the root hair and root surface. Then, these fall off from the root and float on the surface of the nutrient solution or are deposited in the nutrient solution, and these white insoluble crystals were identified using XRD to be Mn phosphate, also called metaswitzerite, with the molecular formula (Mn,Fe)_3_(PO_4_)_2_·4H_2_O. Crystals of Mn phosphate were observed to appear on the root surface in a time sequence negatively correlated with the number of leaves treated with 5 mM Mn. Under the treatment of Mn^2+^ at different concentrations, 1~5 mM, the appearance of Mn crystals and an increase in solution pH were also observed, and the appearance of Mn crystals was positively correlated with the concentration of Mn^2+^ in time series. Compared with the control, the pokeweeds treated without P or Fe accumulated more Mn in their leaves. After further culturing, the plants without P treatment soon (about 15 days) developed the typical symptoms of Mn phytotoxicity, such as young leaf necrosis and leaf chlorosis, while the control plants did not show toxic symptoms.

## Figures and Tables

**Table 1:** Mn accumulation in the leaves of pokeweed treated with 5 mM Mn for 9 d.

Treatment	Mn Content (mg kg^−1^, DW)
Control	3488.7 ± 314.9
−P	4753.3 ± 123.5 **
−Fe	5604.3 ± 78.0 **

** indicates a significant difference at the 1% level compared with control group. −P (or −Fe) indicates the nutrient solutions contain no phosphate (or Fe). Values shown are means ± SD (n = 3).

## Data Availability

Data are contained within the article.
